# Optimizing Mycophenolate Therapy in Renal Transplant Patients Using Machine Learning and Population Pharmacokinetic Modeling

**DOI:** 10.3390/medsci13040235

**Published:** 2025-10-20

**Authors:** Anastasia Tsyplakova, Aleksandra Catic-Djorđevic, Nikola Stefanović, Vangelis D. Karalis

**Affiliations:** 1 Department of Pharmacy, School of Health Science, National and Kapodistrian University of Athens, 15784 Athens, Greece; antsyplakova@pharm.uoa.gr; 2 Department of Pharmacy, Faculty of Medicine, University of Nis, 18000 Nis, Serbia; aleksandra.catic@medfak.ni.ac.rs (A.C.-D.); nikola.stefanovic@medfak.ni.ac.rs (N.S.)

**Keywords:** immunosuppressive therapy, machine learning, population pharmacokinetic modeling, simulations, mycophenolic acid, dosage optimization, renal transplantation

## Abstract

Background/Objectives: Mycophenolic acid (MPA) is used as part of first-line combination immunosuppressive therapy for renal transplant recipients. Personalized dosing approaches are needed to balance efficacy and minimize toxicity due to the pharmacokinetic variability of the drug. In this study, population pharmacokinetic (PopPK) modeling and machine learning (ML) techniques are coupled to provide valuable insights into optimizing MPA therapy. Methods: Using data from 76 renal transplant patients, two PopPK models were developed to describe and predict MPA levels for two different formulations (enteric-coated mycophenolate sodium and mycophenolate mofetil). Covariate effects on drug clearance were assessed, and Monte Carlo simulations were used to evaluate exposure under normal and reduced clearance conditions. ML techniques, including principal component analysis (PCA) and ensemble tree models (bagging and boosting), were applied to identify predictive factors and explore associations between MPA plasma/saliva concentrations and the examined covariates. Results: Total daily dose and post-transplant time (PTP) were identified as key covariates affecting clearance. PCA highlighted MPA dose as the primary determinant of plasma levels, with urea and PTP also playing significant roles. Boosted tree analysis confirmed these findings, demonstrating strong predictive accuracy (R^2^ > 0.91). Incorporating saliva MPA levels improved predictive performance, suggesting that saliva may be a complementary monitoring tool, although plasma monitoring remained superior. Simulations allowed exploring potential dosing adjustments for patients with reduced clearance. Conclusions: This study demonstrates the potential of integrating machine learning with population pharmacokinetic modeling to improve the understanding of MPA variability and support individualized dosing strategies in renal transplant recipients. The developed PopPK/ML models provide a methodological foundation for future research toward more personalized immunosuppressive therapy.

## 1. Introduction

Mycophenolic acid (MPA) is part of the first-line immunosuppressive treatment combination, often including calcineurin inhibitors (CNIs) and corticosteroids. MPA exhibits a complex mechanism of action by inhibiting inosine monophosphate dehydrogenase (IMPDH). This inhibition specifically affects the de novo pathway of purine synthesis, resulting in cytostatic and reversible effects on T and B lymphocytes and preventing transplant rejection.

MPA is administered as mycophenolate mofetil (MMF) or as enteric-coated mycophenolate sodium (EC-MPS). MMF is a prodrug that is rapidly hydrolyzed to MPA, whereas EC-MPS releases MPA in the intestine, following delayed absorption. MMF and EC-MPS are not bioequivalent; conversion to equimolar doses is necessary before comparing the MPA content [1,2,3,4,5]. MPA has a large volume of distribution and is highly protein-bound (>98%) to albumin. It is metabolized by glucuronyl transferases primarily to the inactive metabolite Mycophenolic Acid Glucuronide (MPAG). When excreted in bile, it undergoes enterohepatic recirculation (EHR) and hydrolysis to MPA, causing secondary peaks. MPA mainly undergoes renal elimination as MPAG (over 60%) and only 3% as unchanged MPA [1,4].

MPA exhibits high pharmacokinetic (PK) variability, most often due to genetic polymorphisms, interactions with co-medications such as cyclosporine (a CNI typically part of immunosuppressive therapy combinations), hepatic and renal function, as well as EHR. The development of tools and approaches to support personalized treatment is crucial. Low MPA levels are associated with an increased risk of graft rejection, while high levels increase toxicity, such as gastrointestinal and hematological issues [1,2,3,4,6].

Hepatic and renal function are significant factors in the dosage optimization process, particularly in cases of impaired clearance [7]. Despite the recommendation for increased monitoring, no specific dosage adjustments are currently advised for MPA in renal transplant patients with reduced glomerular filtration rate (GFR) or hepatic insufficiency [5], which may lead to an increased concentration of the MPA.

The most reliable marker of MPA exposure is the area under the concentration curve from zero to 12 h (AUC_0–12_) between 30 and 60 μg·h/mL, but its estimation requires multiple blood samples. Since only the free form of MPA is pharmacologically active and it can be detected in saliva, the possibility of using saliva instead of plasma concentrations for monitoring has been suggested in the literature. Furthermore, saliva collection is a non-invasive method that can increase patient convenience and allow for multiple samples to be taken, thereby enabling better monitoring of therapy. However, the limitations of this method should be considered, such as the availability of sample volumes, low analyte concentrations or potential interaction with food or medication residuals [8,9].

To control the different levels of MPA variability, population pharmacokinetic (PopPK) modeling has been applied to describe PK processes like absorption, distribution and elimination and explore the impact of different factors on MPA behavior [10]. PopPK is a significant tool in therapy optimization because it enables real-time dose adjustments with specific data characteristics, leading to improved clinical outcomes [11]. Nevertheless, it also has some limitations, such as relying on oversimplified PK assumptions and failing to fully describe the variability and complexity of the drug’s responses in patients [7].

An entirely different approach is to apply machine learning (ML), which has emerged as a powerful tool for optimizing drug dosing by identifying complex relationships between patient characteristics and PK parameters. ML techniques, such as ensemble learning, neural networks, and decision trees, can process large datasets to uncover patterns influencing drug metabolism, clearance, and response. These methods enhance predictive accuracy, enabling personalized dose adjustments based on real-time patient data. By continuously learning from new clinical inputs, ML-driven models enhance treatment precision, minimize adverse effects, and facilitate individualized pharmacotherapy [12,13]. However, despite their predictive power, ML models often lack pharmacological interpretability, as they describe empirical relationships rather than underlying biological mechanisms. Combining both (PopPK and ML) approaches allows for a synergistic framework: PopPK ensures physiological plausibility and parameter transparency, whereas ML enhances predictive accuracy and covariate identification [14]. This integration could enable more precise, personalised dosing approaches and offer a better insight into the factors influencing MPA exposure [15,16].

In this study, this innovative approach was followed to investigate and optimize MPA pharmacotherapy in renal transplant patients. It aimed to develop and validate two distinct PopPK models for the EC-MPS and MMF formulations. ML was applied to investigate all available data thoroughly and to identify and quantify the clinical and biochemical parameters that influence MPA concentrations. The goal was to uncover potential relationships between MPA levels and key patient factors. Through PopPK and ML analyses, the relationship between MPA plasma and saliva concentrations was investigated to determine whether saliva MPA levels could serve as a monitoring marker in MPA therapy. The last part of this work focused on precision medicine and particularly on the development of novel dosage regimens that would be appropriate for adult renal transplant patients with either liver or kidney insufficiency.

## 2. Materials and Methods

### 2.1. Study Design and Participants

This cross-sectional study was conducted in renal transplant patients from the nephrology clinic, University Clinical Centre of Nis, Serbia, over a six-month period starting in September 2018. Ethical approval was obtained from the respective institutional review boards, and the study was conducted under the Declaration of Helsinki, with approval from the Ethics Committee of the Faculty of Medicine, University of Nis (No: 12–10 580-2/6, dated 9 October 2018).

Seventy-six patients who had undergone renal transplantation participated after giving written consent prior to analysis were randomly screened. The inclusion criteria required that participants were adult renal transplant recipients who had been at least three months post-transplant, had stable graft function and were on an immunosuppressive regimen that included MPA, tacrolimus, and low-dose prednisone [17]. Furthermore, participants must have had no clinically significant hypoalbuminemia, indicated by serum albumin levels above 25 g/L. The exclusion criteria included patients with active infections, significant comorbidities such as liver disease or heart failure, those on other immunosuppressive drugs that could potentially affect MPA pharmacokinetics, and those with unstable graft function or recent graft rejection within the prior three months. Two oral formulations of MPA were used: MMF (Cellcept^®^, Roche Registration GmbH, Grenzach-Wyhlen, Germany), 500–2000 mg per day administered twice daily, and EC-MPS (Myfortic^®^, Novartis Ireland Limited, Dublin, Ireland), 360–1440 mg per day administered twice daily [2,3]. To compare the two formulations, MMF doses were adjusted by a conversion factor of 0.72 since EC-MPS has a bioavailability of 72%, compared to the MMF tablet 94% [5,18].

### 2.2. Data Collection

Each participant was monitored for six months, during which MPA levels were measured monthly in plasma, while saliva levels were assessed only during the initial visit [19]. Some patients had only one plasma measurement, whereas others had multiple measurements due to more frequent follow-ups. In all cases, trough levels (C_0_) were measured once a steady state had been reached to ensure an accurate evaluation of drug exposure over time. Blood and saliva samples were collected at each participant’s first follow-up visit to determine trough concentrations before the morning dose [8,9]. The sample volumes collected from each patient were 3 mL of blood and 2 mL of saliva. Patients were advised to avoid eating, drinking, and brushing their teeth for 15 min before saliva collection sampling. After collection, whole blood and saliva samples were centrifuged for 15 min at 1522× *g* and 22 °C, then stored at −80 °C until analysis [9]. Blank samples were also collected from healthy volunteers under the same pre-analytical conditions as patient samples. These samples were used exclusively as negative controls to confirm the absence of MPA and to check for any analytical interferences. No demographic or clinical data were gathered for these volunteers, as they were not involved in the pharmacokinetic study analysis. On the other hand, for each patient, demographic and clinical parameters, including age, sex, post-transplantation time, and the type of donor transplant (living or deceased), were recorded. In addition, biochemical markers such as renal function parameters (urea, creatinine) and hematological parameters (white blood cells (WBCs), red blood cells (RBCs), hematocrit (Ht), hemoglobulin (Hb), platelets [PLT]) were also analyzed [7,10]. These parameters were selected based on their potential influence on MPA PKs and clinical relevance in transplant patient management.

### 2.3. Population Pharmacokinetics Modeling

PopPK modeling was performed using Monolix™ 2023R1, applying nonlinear mixed-effects (NLMEs) modeling to analyze MPA’s pharmacokinetics. The objective was to identify key factors influencing MPA pharmacokinetics and develop two distinct PopPK models: Model 1 for EC-MPS formulation and Model 2 for MMF formulation [19,20].

PK analysis was conducted using MPA plasma concentration-time (C-t) data collected after the administration of EC-MPS or MMF. The number of compartments describing drug disposition was initially examined by testing one-, two-, and three-compartmental models, while also exploring potential delays in absorption by including lag times or transit compartments [20,21]. The key PK parameters estimated included volume of distribution (V), which represents the apparent volume in which the drug is distributed, clearance (CL), which is defined as the fraction of V cleared of the drug per unit of time, and the absorption rate constant (ka), indicating the rate at which the drug enters the bloodstream [22,23].

PK parameters were assumed to follow a lognormal distribution, and an exponential model was used to account for inter-individual variability (IIV). Inter-occasion variability (IOV) was also incorporated, treating each subsequent visit as a separate occasion. The equations used for the individual models, including the effect of continuous covariates (1) and categorical (2), are as follows:

(1) $$\log\left(P_{i}\right)=\log\left(P_{pop}\right)+\;betaC*\log\left(\frac{C_{i}}{C_{mean}}\right)+\;n_{i}\;+\;k_{ij}$$

(2) $$log(P_{i})=log(P_{pop})+betaG*G_{i}+n_{i}+k_{ij}$$

where

- *P_i_* is the PK parameter value for the *ith* subject;
- *P_pop_* is the population mean estimate of the parameter;
- *betaC* is the effect of the continuous covariate Ci for the *ith* subject on the PK parameter;
- *betaG* is the effect size of the categorical covariate Gi for the *ith* subject on the PK parameter;
- *C_mean_* is the mean value of the covariate for normalization;
- *n*~*N*(0, *ω*^2^) represents the interindividual variability (IIV). Thus, for the *ith* subject, the IIV is *n_i_*;
- *k*~*N*(0, *γ*^2^) represents the interoccasion variability (IOV). Thus, for the *ith* subject at the *jth* visit the IOV is *k_ij_*.

Residual unexplained variability, including interindividual variability, was described using additive (3), proportional (4), and combined (5) error structures, which were evaluated to optimise the model’s performance, as shown below.

(3) Cobs=Cpred+a∗ε1

(4) Cobs=Cpred×(1+b∗ε2)

(5) Cobs=Cpred ∗ (1+b∗ε2)+a ∗ε1

where

- *Cobs* represents the observed concentration;
- *Cpred* represents the model-predicted concentration;
- *a* is the additive component reflecting a constant deviation independent of concentration;
- *b* is the proportional component reflecting variability that increases with concentration;
- ε_1_ and ε_2_ ~N(0, 1) are the random variables that represent the unexplained deviation between *Cpred* and *Cobs*.

The most appropriate residual error structure was selected based on the lowest objective function value (OFV), inspection of goodness-of-fit plots, and homogeneity of weighted residuals across the predicted concentration range.

Since only one measurement per occasion (trough levels) was available for each patient, a stepwise procedure was followed. Initially, one PK parameter was freely estimated. In contrast, the others were fixed to literature values or obtained via maximum a posteriori estimation. Once a robust estimate was achieved, additional parameters were allowed to vary. Various estimation sequences were tested, selecting the best-fitting and physiologically sound model [24,25,26].

To refine the models, the effect of several covariates on PK parameters was evaluated using stepwise forward and backward selection. The covariates analyzed included demographic factors such as age, daily dose, and PTP, biochemical markers including renal function parameters (urea and creatinine), and hematological parameters (white blood cells, red blood cells, and platelets), as well as transplant-related factors such as the type of donor (live vs. deceased), as seen in Table 1. The Pearson correlation test and one-way ANOVA assessed continuous and categorical covariates, respectively. The Wald test determined whether a covariate significantly contributed to the variation in pharmacokinetic parameters, with a significance level set at 5%.

Covariate selection was based on multiple criteria, including the decrease in the −2 log likelihood (−2LL) value, indicating an improved model fit, the precision of parameter estimates assessed by relative standard error (RSE%), the reduction in IIV to improve model robustness, and the physiological relevance of the selected covariates, ensuring a meaningful biological effect on drug pharmacokinetics. These methods ensured the development of robust and individualized PopPK models, providing a data-driven approach to optimizing MPA therapy in renal transplant patients.

#### Model Validation

The final model was chosen based on statistical and graphical goodness-of-fit criteria, which helped identify any potential biases or issues in the structural model. The precision of parameter estimates was evaluated using RSE%, and inter- and intra-individual variability were assessed to quantify variation between patients and within patients over time. To compare and select the optimal model, numerical statistical significance criteria such as log-likelihood, the Akaike Information Criterion (AIC), and the Bayesian Information Criterion (BIC) were used to select non-hierarchical models. Graphical validation involved comparing observed plasma concentrations with predicted concentrations, providing a visual assessment of the model’s predictive and descriptive ability. Goodness-of-fit was further evaluated using plots of weighted residuals vs. concentrations or time, as well as individual fits. Additionally, visual and numerical predictive check (VPC and NPC) charts were created using 1000 Monte Carlo simulations with 90% confidence intervals to evaluate the model’s predictive performance, stability, and robustness. The final model was selected based on its ability to explain observed data effectively, ensuring the smallest relative standard error and lowest inter-individual variability.

### 2.4. Machine Learning Techniques

ML techniques are powerful tools in advancing the predictive accuracy of MPA plasma levels by identifying key factors that influence these levels. ML methods include many algorithms, enabling researchers to select the most appropriate approach to extract meaningful findings from complex datasets and uncover intricate relationships between MPA concentrations, clinical parameters, and patient-specific characteristics. In this context, various ML approaches, such as Principal Component Analysis (PCA), Support Vector Machines (SVMs), Artificial Neural Networks (ANNs), Random Forest (RF), and ensemble learning techniques (like boosted and bagged trees), were used. However, due to the limited number of observations, the dataset was insufficient to support reliable ANN development. SVMs were also tested, but did not demonstrate satisfactory predictive performance compared with the selected ensemble methods. After an extensive search, the two ensemble methods and PCA yielded the best descriptive and predictive performance [27,28,29]. For this reason, these methods are only presented below.

#### 2.4.1. **Unsupervised Machine Learning:** Principal Component Analysis

PCA is an unsupervised ML method that reduces high-dimensional datasets while preserving as much information as possible [28]. PCA transforms a set of potentially correlated variables into a new set of uncorrelated variables, known as principal components [30]. The components are ranked according to the amount of variance they explain and the information they include. Principal components are less interpretable and lack real meaning, as they are constructed as linear combinations of the initial variables. In this way, data becomes easier to handle and reveals patterns that might otherwise be unnoticed [31]. In this study, PCA was applied to explore the correlations between multiple factors, including MPA dose, urea levels, PTP, MPA saliva concentrations, patient age, haematological parameters (e.g., red blood cell count, haemoglobin), and plasma MPA levels.

#### 2.4.2. Ensemble Methods

Ensemble tree modeling is a supervised ML method that follows a different approach by constructing many decision trees during training and combining their outputs to produce a more accurate and stable prediction [28]. Each tree is built on a random subset of the data and features, which helps avoid overfitting [30]. This technique captures non-linear relationships and identifies the most influential predictors of a target variable, such as MPA plasma levels [13,32]. This study employed boosted tree regression to predict MPA concentrations based on multiple predictor variables. Boosting iteratively improves the model by correcting errors made by previous iterations, often leading to higher accuracy [27]. This method was selected due to its ability to handle nonlinear relationships and interactions between features, as well as analyze even moderately sized datasets like ours [12,25].

#### 2.4.3. Model Implementation and Software

Implementing these ML techniques required a structured workflow executed using Python version 3.10.8. Several specialized libraries were used: sklearn (scikit-learn) provided tools for PCA and model evaluation [28]. For boosted tree analysis, a gradient boosting algorithm was employed after importing XGBoost for high-performance prediction, as most variables are continuous [13]. Additionally, MLxtend supported further ensemble strategies and feature selection [32]. Data preprocessing and cleaning were first performed using the pandas library, followed by preprocessing with scikit-learn to normalize variables and ensure consistency. The dataset was examined for completeness and consistency; no missing values or outliers were identified, and therefore no imputation was required [31]. Feature selection was then performed, where the most relevant predictors (e.g., MPA dose, age, or urea levels) were identified based on their statistical and clinical significance [12].

In the case of boosted tree analysis, the regression model was trained using an 80% training split and a 20% testing split of the dataset [14]. To further evaluate model generalizability and minimize the risk of overfitting, 5-fold cross-validation was performed, allowing each data subset to serve as a validation set in turn. Hyperparameter tuning (using Grid search, random search, and Bayesian optimization) was a critical step involving adjusting settings like the number of trees or the learning rate in boosting models to balance underfitting and overfitting [27]. Model performance was assessed using metrics such as mean squared error (MSE), which quantifies prediction error, and coefficient of determination (R^2^), which measures how well the model explains the variance in MPA levels [25,32]. To interpret and evaluate the model, a SHAP (SHapley Additive Explanations) Summary Plot was used to visualize the contribution of each feature to individual predictions [13].

Regarding PCA, the number of principal components was determined using the explained variance ratio and the scree plot, ensuring that sufficient information was preserved in the first two principal components [30]. Loading plots were constructed to visualize feature vectors in the space of the first two principal components and detect clustering or patterns in the data [28]. The direction and length of vectors indicate the contribution of each feature to the principal components (PC1 and PC2) and their associations with each other [12]. Two PCA models were developed: one included MPA plasma levels, demographic, and clinical parameters, and the other also included saliva levels.

Overall, in this study, PCA was used to explore the data structure, while ensemble methods were applied to enhance predictive accuracy and interpretability [25,31].

### 2.5. Monte Carlo Simulations

Monte Carlo simulations based on the EC-MPS-developed PopPK model were performed. The PK profiles of a thousand subjects were simulated, consisting of new individual parameter values derived from the model’s previously defined distributions of the population values and the covariates [33]. Dosage regimens within the reference range of 360 mg and 720 mg twice daily were simulated for renal transplant patients, using a mean population overall clearance of healthy subjects, with reductions of 50% and 75% [7]. The aim was to simulate the repeated oral administration of EC-MPS until it reached a steady state; dosage regimens for repeated administration at prolonged intervals were simulated [21]. After performing model-based simulations, the predicted concentration–time profiles were used to calculate the MPA AUC_0–12_ values in Datanalix^™^ (Monolix^®^ 2023R1, Lixoft, Antony, France), which were then compared with the established therapeutic reference range of approximately 30–60 μg·h/mL [3]. Based on these simulations, new dosage regimens were explored to achieve target exposure levels in patients with impaired renal or hepatic function [7]. The simulated concentration-time plots are presented with percentiles based on the data from the simulated individuals [33]. For clarity, the graphs display the 5th and 95th percentiles, along with a 90% confidence interval (shaded area), and the median of the observations [21]. For simplicity and consistency in this study, the evaluation of concentration levels across different dosage regimens was based on the median variation. Monte Carlo simulations were conducted using Simulx^™^ (Monolix^®^ 2023R1, Lixoft, Antony, France).

## 3. Results

The results from the PopPK modeling and ML analyses provide valuable insights into MPA PKs and highlight key factors influencing drug concentrations.

### 3.1. Population Pharmacokinetic Modeling

Two PopPK models were developed and validated: one for the EC-MPS formulation (Model 1) and one for the MMF formulation (Model 2). Both models were based on plasma concentration data and assumed a one-compartment model with first-order absorption and elimination kinetics. The models were validated using statistical and graphical methods, including plots of observed vs. predicted concentrations, individual fits, and numerical predictive check plots.

#### 3.1.1. EC-MPS Model (Model 1)

The parameter estimates for the EC-MPS formulation model (Table 2) indicated that the absorption rate constant (kapop) was 0.18, with an RSE of 15.7%. The volume of distribution (Vpop) was estimated at 192.42 L (RSE = 18.8%), and the clearance (Clpop) was 9.3 L/h (RSE = 8.34%). The absorption rate constant (kapop) and the volume of distribution (Vpop) were estimated using maximum a posteriori estimation. At the same time, clearance was determined via maximum likelihood estimation with initial values sourced from the literature. The potential influence of the examined covariates on the PK parameters was also quantified. The post-transplantation time (PTP) and total daily dose (TDD) in mg were significant covariates for clearance, exhibiting RSE values of 24.7% and 19.2%, respectively. Below, the equations describing the individual models of PK parameters are presented:

(6) $$log(k_{ai})=log(0.18)+n_{IDk_{ai}}+n_{IOVk_{aij}}$$

(7) $$log(V_{i})=log(192.42)+n_{IDk_{ai}}+n_{IOVV_{ij}}$$

(8) $$\log({Cl}_{i})=\log\left(9.3\right)+0.16*\log(\frac{Diff\_in\_months}{67})+0.77*\log(\frac{TDD}{1500})+n_{ID{Cl}_{i}}+n_{IOV{Cl}_{ij}}$$

The random effect standard deviations were estimated for ka [ω(ka)] = 0.36, RSE = 21.2%), V [ω(V) = 0.52, RSE = 38.8%], and Cl [ω(Cl) = 0.27, RSE = 20.9%]. These estimates suggest moderate interindividual variability in drug absorption, distribution, and clearance across individuals. The combined additive and proportional error model, which accounts for residual unexplained variability, was also characterized by parameters “a” and “b” with RSE values of 26% and 24.6%, respectively.

#### 3.1.2. MMF Model (Model 2)

The MMF formulation model yielded similar pharmacokinetic parameter estimates (Table 3). The kapop was 0.23 (RSE = 22.6%), Vpop was 196.43 L (RSE = 28.9%), and Clpop was 9.3 L/h (RSE = 11.2%). The covariates influencing clearance were also time post-transplantation [β(PTP)] and total daily dose [β(TDD)], with RSE values of 21.8% and 23.1%, respectively. Random effect standard deviation estimates were found to be omega_ka = 0.27 (RSE = 26.5%), ω(V) = 0.09 (RSE = 32.4%), and ω(Cl) = 0.32 (RSE = 19.2%), indicating lower variability in drug absorption and distribution in comparison to the EC-MPS formulation model. The proportional error model parameters had RSE values of 37.5% for “b”, representing the residual variability in the model.

The following equations illustrate the models for individual PK parameters:

(9) $$Log(k_{ai})=log(0.23)+n_{IDk_{ai}}+n_{IOVk_{aij}}$$

(10) $$Log(V_{i})=log(196.43)+n_{IDV_{i}}+n_{IOVV_{ij}}$$

(11) $$Log({Cl}_{i})=\log(9.3)+0.33*\log(\frac{Diff_{in_{months}}}{21})+1.27*\log(\frac{TDD}{1500})+n_{ID{Cl}_{i}}+n_{IOVClij}$$

**Figure 1:**
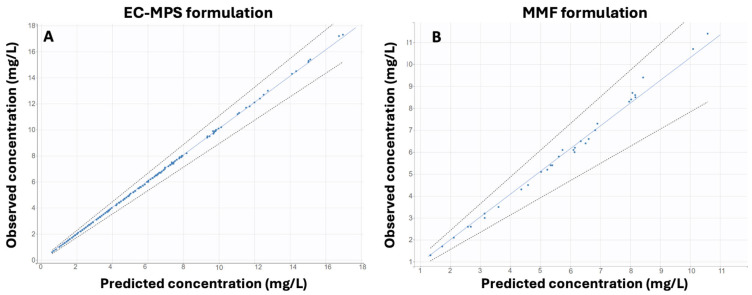
Observed vs. individual predicted concentrations for the final population pharmacokinetic (PopPK) models of Mycophenolic Acid (MPA): (**A**) Enteric-Coated Mycophenolate Sodium (EC-MPS) formulation and (**B**) Mycophenolate Mofetil (MMF) formulation.

**Figure 2:**
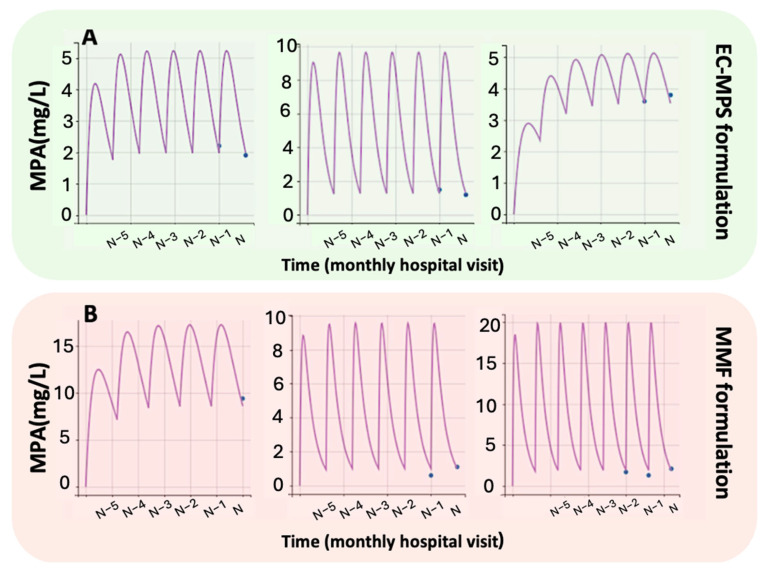
Individual model fittings to the experimental MPA plasma concentration–time data for (**A**) Enteric-Coated Mycophenolate Sodium (EC-MPS) and (**B**) Mycophenolate Mofetil (MMF) formulations. Due to space limitations, only the first three representative subjects per formulation are shown. Time points correspond to consecutive monthly hospital visits.

**Figure A1:**
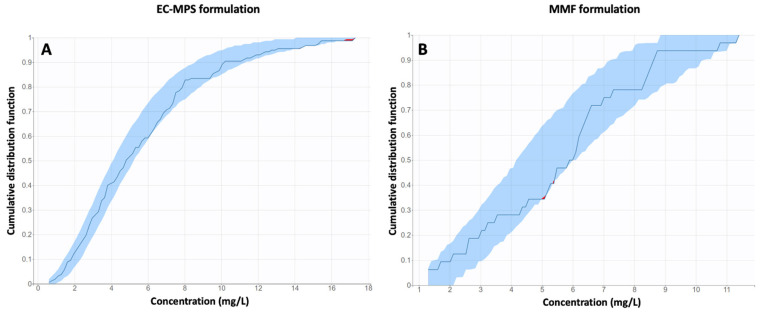
Numerical predictive check plots for the two developed population pharmacokinetic models describing MPA plasma levels. Administration of the (**A**) Enteric-Coated Mycophenolate sodium (EC-MPS) formulation, (**B**) Mycophenolate mofetil (MMF) formulation.

Both models were validated using individual predicted vs. observed concentrations data (Figure 1) and individual fitting data plots (Figure 2), illustrating a good agreement between predicted and observed plasma MPA concentrations. The NPC plots (Figure A1) also showed that both models performed well in predicting drug concentrations with acceptable precision in individual predictions.

### 3.2. Machine Learning Analysis

To further explore the factors influencing MPA levels, ML techniques, including boosted tree analysis and PCA, were applied to identify key features contributing to plasma MPA concentrations.

#### 3.2.1. Boosted Trees

**Figure 3:**
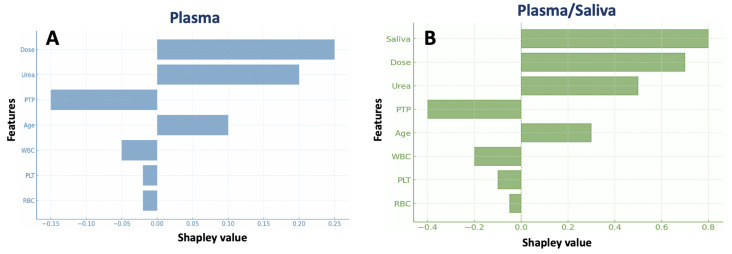
Shapley summary plots for the boosted trees regression analysis showing the contribution of each feature to the plasma MPA levels, without (panel (**A**)) and with (panel (**B**)) saliva MPA data.

Boosted tree analysis, as visualized through a SHAP summary plot, enabled the quantification of the importance of clinical and biochemical factors in predicting plasma MPA concentrations. For the plasma-only model (Figure 3A), the TDD was identified as the most influential factor, followed by urea levels and PTP. Dose and urea were associated with positive Shapley values, indicating their strong positive contribution to MPA concentration predictions. At the same time, PTP showed a negative influence, suggesting that drug concentrations decrease over time after the transplantation. Other factors, such as age, WBC, PLT, and RBC, had relatively minor contributions to the model’s predictive power. The model achieved a high predictive performance (R^2^ > 0.91; 95% CI ≈ ±0.1), indicating stable generalization across the dataset.

For the plasma and saliva model **(**Figure 3B), the SHAP plot revealed that saliva concentration made the most substantial contribution to MPA concentration predictions, followed by dose, urea, and PTP. This indicates that incorporating saliva data improved model performance, as saliva concentrations provide additional predictive power beyond plasma measurements.

#### 3.2.2. Principal Component Analysis

**Figure 4:**
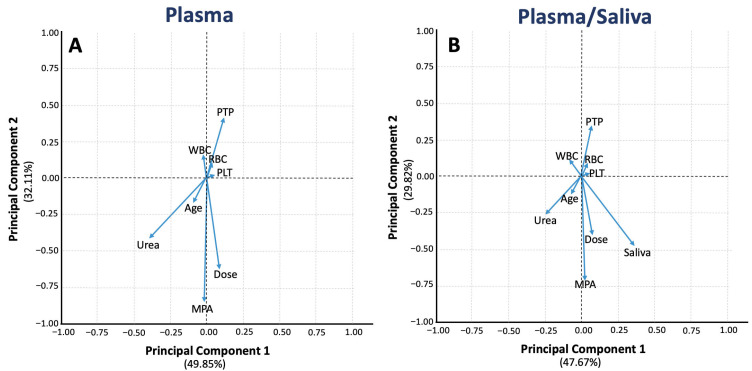
Principal component analysis showing the loading plots without (panel (**A**)) and with (panel (**B**)) saliva data. The features shown refer to MPA levels, Age, Dose, Urea, PTP, RBC, PLT, and WBC vectors. Key: Age, age of participants; Dose, total daily dose of MPA; MPA, mycophenolic acid; PLT, platelets; PTP, post-transplantation period; RBC, red blood cells count; Saliva, MPA saliva levels; Urea, urea plasma levels; WBC, white blood cells count.

PCA was applied to reduce the high-dimensional dataset and uncover relationships among clinical factors and drug concentrations. The loading plot for the plasma model (Figure 4A) revealed strong positive correlations between MPA plasma concentrations and both dose and urea levels. PTP was negatively correlated with plasma drug concentrations, suggesting a decrease in MPA levels as time progresses post-transplantation. Hematological parameters (WBC, RBC, and PLT) formed a cluster, indicating their shared influence on variability within the plasma dataset, likely reflecting broader physiological changes after transplantation and hematological toxicity. When saliva concentrations were included in the analysis (Figure 4B), MPA plasma concentrations remained strongly correlated with dose and urea, but saliva concentrations emerged as an additional important factor. Saliva was positioned along PC1, indicating that it is a key source of variability in the dataset. PTP, WBC, RBC, and PLT kept their clustering, and urea and age showed similar relationships to those observed in the plasma-only model, with slight influences from the addition of the saliva vector.

These findings suggest that while plasma concentrations remain the gold standard for therapeutic monitoring, saliva concentrations consistently and meaningfully reflect MPA exposure. Their strong correlation with plasma levels indicates that saliva could serve as a feasible, non-invasive alternative or supplement for patient follow-up, particularly when blood sampling is impractical or needs to be minimized. However, the clinical applicability of saliva monitoring should be interpreted with caution, as further validation in larger, longitudinal studies is necessary before integrating it into routine practice.

### 3.3. Impact of Renal Function on MPA Levels

**Figure 5:**
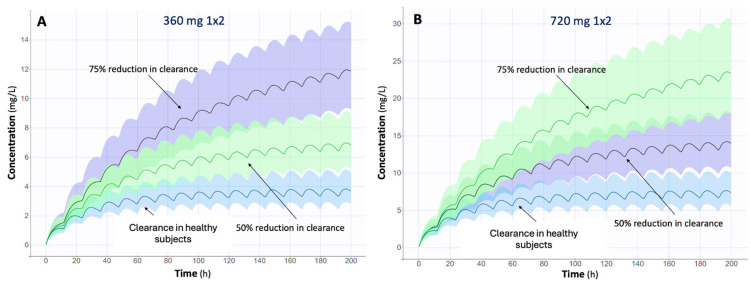
Simulated mycophenolate plasma concentrations (mg/L) vs. time (hours) profiles of mycophenolic acid (MPA) after initiation of treatment, following administration of (**A**) 360 mg twice daily and (**B**) 720 mg twice daily dosing regimens under three conditions (clearance in healthy subjects, 50% reduced clearance, and 75% reduced clearance).

One of the key objectives of this study was to evaluate how impaired renal clearance affects MPA PKs. Simulation analyses were performed to assess the C-t profiles of MPA under different clearance conditions for the two dosing regimens officially proposed: 360 mg twice daily (Figure 5A) and 720 mg twice daily (Figure 5B). These simulations were based on the EC-MPS model, which was selected as the most robust due to the larger proportion of data derived from this formulation. Three conditions were examined: clearance in healthy subjects, 50% reduced clearance, and 75% reduced clearance. For this study, the clearance in healthy subjects was defined as the mean population clearance (Cl_pop = 9.3 L/h) estimated from the developed PopPK model (Table 2).

These simulations showed that as clearance decreased, MPA concentrations increased due to slower drug elimination. This effect was more pronounced at higher doses, with the 720 mg twice-daily regimen showing a significant increase in MPA concentrations in patients with impaired renal function. In the case of 75% reduced clearance, drug concentrations were substantially higher, potentially leading to an increased risk of toxicity. These results underline the importance of individualized dosing adjustments, especially for patients with impaired renal function, to maintain therapeutic MPA concentrations and avoid drug accumulation.

**Figure A2:**
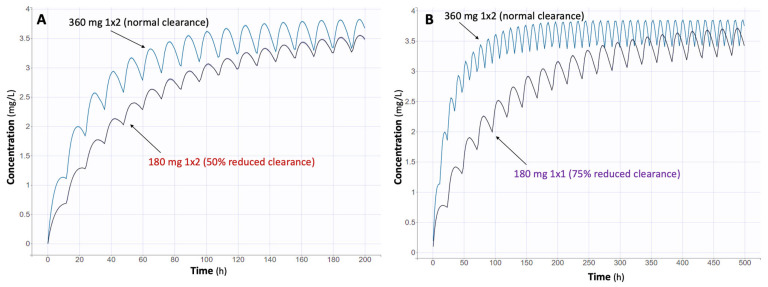
Simulated dosage regimens of 360 mg enteric-coated mycophenolate sodium formulation administered twice daily in patients with normal clearance, along with simulated adjusted dosage regimens of 180 mg 1 × 2 and 180 mg 1 × 1 for cases of 50% reduced clearance (**A**) and 75% reduced clearance (**B**), respectively.

**Figure A3:**
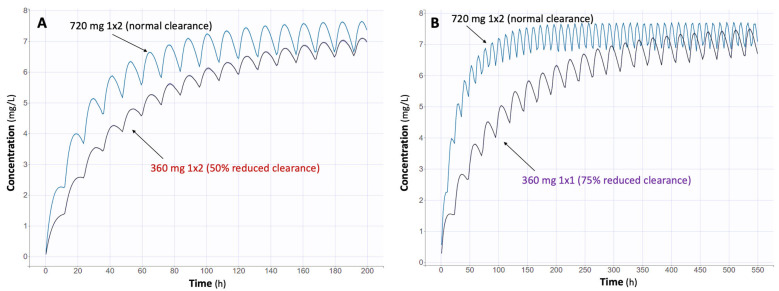
Simulated dosage regimens of 720 mg enteric-coated mycophenolate sodium formulation administered twice daily in patients with normal clearance, along with simulated adjusted dosage regimens of 360 mg 1 × 2 and 360 mg 1 × 1 for cases of 50% reduced clearance (**A**) and 75% reduced clearance (**B**), respectively.

Simulations also suggested potential dosing adjustments for patients with reduced overall clearance. For the 360 mg twice-daily regimen, the recommended adjusted doses for 50% and 75% S2-reduced clearance were 180 mg twice daily and 180 mg once daily, respectively (Figure A2). For the 720 mg twice-daily regimen, the adjusted doses for reduced clearance were 360 mg twice daily and 360 mg once daily (Figure A3). These proposed regimens aim to maintain MPA concentrations within the therapeutic window while minimizing the risk of toxicity in patients with impaired renal function.

## 4. Discussion

This study focused on optimizing MPA therapy for renal transplant recipients by using both PopPK modeling and ML methods. Additionally, this study examined the utility of noninvasive saliva monitoring as a potential substitute for plasma MPA concentration monitoring, revealing promising implications for clinical practice. The significant findings from this study are divided into two main categories: (a) the development and validation of two PopPK models as well as simulations for new dosage regimens in the case of renal and hepatic insufficiency, and (b) the use of ML techniques to identify important factors affecting MPA plasma levels.

Most PopPK models for MPA utilize a two-compartment structure with lag time in absorption and first-order kinetics, often incorporating linear protein binding and EHC parameters. In this study, a one-compartment model with first-order absorption and elimination for both MMF and EC-MPS was ultimately selected, as only a single trough level measurement was available at steady state for each occasion. This limited dataset made it challenging to estimate more complex PK parameters. Lag time in absorption was not explicitly identified. Still, the low absorption rate constants (lower than those estimated for the literature MPA PopPK models) for both models (Table 2 and Table 3) suggest a delay in absorption. Interestingly, the absorption rate constant for EC-MPS is similar to that of MMF but slightly lower, reflecting the delayed absorption of the enteric-coated formulation.

MPA pharmacokinetics exhibit considerable IIV, making PopPK crucial for dosage optimization alongside TDM or limited sampling strategies [19,22]. Studies highlight significant IIV in MPA PK, underlining the need for individualized dosing to prevent adverse effects and graft rejection [11]. MPA plasma concentrations vary among renal transplant patients, influenced by renal function, age, and concomitant medications [17]. However, some studies have reported that demographic factors such as sex, age, and body weight may not significantly affect MPA pharmacokinetics [34]. This variability is reflected in PK parameters reported across published MPA PopPK models, as also demonstrated by a recent systematic review and external evaluation by Gao et al. (2025), which highlighted considerable between-study variability and the need for external validation in diverse populations [35].

In our study, the PK parameter values for the developed models are similar, as they describe the PK of the same active substance (i.e., MPA) after absorption. The findings align with most PopPK studies and the Summary of Product Characteristics (SmPC)/US Prescribing Information (USPI) [1,2,3,4]. The half-life time of MPA from both EC-MPS and MMF formulations is about 14.5 h, within the reference range of 17.9 ± 6.5 h for MMF and 8 to 16 h for EC-MPS [5].

Both models demonstrated that PTP has a significant effect on clearance, as documented in the literature [24,36,37]. Most studies examine the early post-transplantation period, which lasts three months and is characterized by impaired renal function, suppressed metabolism of MPA, and higher immunosuppressant doses. Specifically, during the first days or weeks after transplant, a decrease in total MPA exposure is observed compared to 1 to 6 months post-transplant, which can be attributed to temporary renal dysfunction leading to MPAG accumulation and competition with MPA for albumin binding sites. The increase in the free MPA fraction enhances clearance, thereby lowering total MPA AUC values until renal function and protein binding normalization [38,39]. In this study, these mechanisms are likely stabilized, and acute rejection events have been overcome after three months post-transplant. However, clearance fluctuates over time due to progressively improved renal function that enhances MPAG elimination, lowers albumin binding competition, and boosts hepatic metabolism.

The normalization of hepatic uridine diphosphate glucuronosyltransferase enzyme activity increases MPA glucuronidation, while concomitant immunosuppressive therapy, including CNIs, can further influence MPA disposition. Cyclosporine reduces MPA exposure by inhibiting the multidrug resistance-associated protein 2 transporter, which limits the biliary excretion of MPAG and interrupts EHR. In contrast, tacrolimus does not affect this pathway and is associated with higher MPA AUC. Reduced cyclosporine exposure during therapy or switching to tacrolimus after transplantation can restore MPAG excretion and normalize enterohepatic cycling, promoting more efficient MPA elimination. These differences highlight the importance of TDM when transitioning between CNIs. Additionally, alterations in EHR due to gut microbiota or transporter activity may also contribute to variability in MPA pharmacokinetics and enhance clearance time [40,41].

The second significant covariate positively impacting MMF and EC-MPS clearance is the total daily MPA dose, as shown by Veličković-Radovanović et al. 2015 [42]. Since all patients in our cohort were already in the stable post-transplant phase, the recorded doses likely reflected prior individual adjustments made to achieve therapeutic exposure. Consequently, patients receiving higher total daily doses may have required these regimens to compensate for greater clearance, which could explain why the total daily dose appeared as a positively associated covariate with clearance. MPA shows linear, dose-proportional pharmacokinetics from 360 mg to 2160 mg, suggesting clearance increases with dose due to physiological adaptations rather than immediate saturation or dose-dependent metabolic changes. On the other hand, higher MPA doses exceeding the maximum therapeutic dose of 2160 mg per day administered by some participants in our study may increase the free MPA fraction due to saturation of albumin binding, thereby boosting metabolism and clearance.

Additionally, changes in EHC and transporter activity (e.g., MRP2 upregulation) may further promote MPAG elimination and reduce MPA reabsorption [1,2,3,4]. Standard covariates in the literature include age, body weight, albumin, creatinine clearance (CrCl), PTP, immunosuppressive concomitant medication, dose, and gene polymorphisms [43]. In our study, some patients’ characteristics, such as body weight or albumin levels, were unavailable, which prohibited the calculation of creatinine clearance and MPA protein binding, respectively. Since weight information was unavailable, the identified covariate of total daily dose may partly compensate for body weight, given that mycophenolate dosing can be influenced by patient size.

PopPK models are crucial for personalized dosing and therapeutic optimization, particularly in complex cases such as those involving renal transplant patients. While they describe PK processes well, they often make assumptions and can oversimplify real-world PK variability and physiological processes. ML enhances PK modeling by allowing more accurate individualized dosing, thus reducing toxicity and graft rejection risks. Additionally, ML models adapt better than static PopPK models, continuously updating with new patient data. Finally, ML reveals hidden patterns in large datasets, uncovering subtle PK interactions that traditional methods may miss. A few studies have used ML or DL techniques to predict MPA exposure. In 2021, Woillard and colleagues developed and validated extreme gradient boosting ML models for kidney or heart transplants [44]. These models provided accurate MPA AUC_0–12h_ estimations for personalized dose adjustments based on three blood concentrations, outperforming the previously utilized PK approach. Shao K et al. 2022 proposed a joint DL model for estimating MPA exposure in Chinese renal transplant patients [45]. Lastly, Sun et al. (2023) [46] employed the same technique as Woillard et al. 2021 [44] to develop an ML-based model for AUC_0–12h_ prediction, incorporating body weight, age, EHC-related factors, co-administered CNIs, and genetic polymorphisms of drug transporters to enhance the model’s predictive performance in Chinese renal transplant recipients.

This study combined PopPK modeling and ML techniques to enhance MPA therapy in renal transplant recipients. While PopPK modeling offered mechanistic insights into MPA disposition and covariates, ML analysis supplemented these findings by identifying additional, potentially non-linear predictors of MPA levels. Boosted tree regression analysis and PCA identified key predictors of MPA levels and their connections to clinical variables. Both methods pinpointed MPA dose as the primary predictor, which is consistent with MPA PK discussed above. Urea levels and PTP emerged as significant factors predicting MPA concentrations and influencing the variability of the drug, with an inverse relationship observed between PTP and MPA levels, as well as urea. These findings align with the physiological progression following transplantation: although total MPA exposure is initially low during the early post-transplant period due to temporary renal dysfunction and altered protein binding [43,47], levels gradually stabilise and may decrease over time in the late post-transplant phase as renal and hepatic clearance improve, along with the recovery of protein binding and the tapering of immunosuppressive doses. However, additional studies focusing exclusively on the late post-transplant period are warranted to confirm whether clearance continues to increase and MPA exposure decreases as graft and metabolic functions fully normalize.

Unlike PopPK, ML detected that higher urea levels may correlate with increased MPA concentrations. Reduced renal clearance leads to MPAG buildup and enterohepatic recirculation, raising toxicity risks. Bile flow, gut microbiota, and medications may also affect this phenomenon.

The SHAP plots revealed minor negative effects of WBC, RBC, and PLT on MPA levels, while PCA confirmed their inverse relation with MPA dose. MPA may cause dose-dependent leucopenia, anemia, and thrombocytopenia. Haematological insufficiency probably occurs due to reduced erythropoietin production due to prior renal impairment, with renal allograft stabilization taking three months after transplantation [10]. PCA results further indicate that WBC, PLT, and RBC clustering positively correlate with PTP and inversely with urea, suggesting stabilization as kidney function improves over time. In this study, the median values of WBC, PLT, and RBC were within the normal range for both genders, yet fell within the lowest reference range (Table 1). Moreover, all patients were at least three months post-transplant. Although anaemia may occur at any point post-transplant, leucopenia will most likely arise in the early post-transplant phase [48]. The relationships between haematological parameters, MPA dose, and PTP were noted, further illustrating the high sensitivity of ML as a data analysis method. Changes in WBC, RBC, and PLT may also be linked to immune suppression, graft rejection, infections, or the patient’s recovery, all of which fluctuate over time as the patient adjusts to immunosuppressive therapy.

The moderate effect of age on MPA PKs, resulting from the application of ML techniques, highlights the importance of demographic factors in immunosuppressive therapy. Ageing can affect renal function, hepatic metabolism, and body composition, influencing drug PKs. Although age-related changes contribute to variability in MPA levels, their impact is generally moderate compared to renal function and dosing factors. While age does not have a direct effect on renal function or drug dose, it remains significant in optimizing therapy. Elderly patients may require closer monitoring due to a potential decline in renal function or the presence of comorbidities. Thus, as supported by the literature, age is a significant demographic factor that should be considered in personalized dosing strategies, along with renal function and drug dose [49]. Although the ML models demonstrated high predictive performance, the relatively small sample size may increase the risk of overfitting. External validation using independent datasets is required to confirm the generalizability and robustness of the models.

Both renal and hepatic clearance are crucial for the elimination of MPA. Hepatic dysfunction reduces metabolism and alters enterohepatic circulation, thereby prolonging the effects of MPA. Renal impairment leads to MPAG accumulation, increasing free MPA levels and potential toxicity in renal transplant patients [7,49]. As one clearance pathway may compensate for another, this study modelled overall clearance from both elimination routes. For eGFR ≥ 25 mL/min/1.73 m^2^, no dose adjustment is needed, although TDM is recommended for levels below 60 mL/min/1.73 m^2^ [5]. In severe renal impairment (eGFR < 25 mL/min/1.73 m^2^), the initial dosing remains unchanged; however, caution is advised [5]. No specific dose adjustments for hepatic disease are recommended; however, increased monitoring is advised in cases of hyperbilirubinemia or hypoalbuminemia, as these conditions can alter protein binding and potentially elevate free MPA concentrations [21]. Subtherapeutic MPA levels correlate with a higher rejection risk, while toxicity thresholds remain undefined [50]. Using Monte Carlo simulations, this study modeled moderate and severe clearance impairment and proposed individualized dosing regimens to optimize MPA exposure, thereby maintaining therapeutic concentrations while minimizing rejection risk and toxicity in renal transplant patients.

Future validation of proposed dosing regimens should involve prospective clinical studies comparing model predictions with patient outcomes. These could include therapeutic drug monitoring at steady state to verify MPA exposures and assess outcomes like graft function, rejection, and toxicity. Incorporating real-world data would help refine models and support clinical decisions [51]. These simulation-based regimens are exploratory and intended to guide future clinical validation rather than immediate application in practice.

The study on saliva for MPA therapy monitoring is advantageous, as traditional venous sampling is invasive for transplant patients [9]. While saliva concentrations may not provide precise dosing, they can help monitor trends in MPA exposure. Some studies suggest that saliva may replace plasma concentrations, as evidenced by the survey of Alsmadi et al. (2019) involving pediatric renal transplant patients [52]. Catić-Đorđević et al. (2022) noted that saliva monitoring may help manage adverse effects without requiring plasma comparisons [53]. Ferreira et al. 2019 support saliva monitoring [54], but research with thirteen kidney transplant patients highlights the need for further studies. Other studies have shown a probable weak relationship between plasma and saliva MPA levels in kidney transplant patients, indicating that saliva is an inadequate biomarker for TDM [8,9]. In our study, ML enabled the exploration of combined plasma and saliva data, revealing that saliva concentration had the strongest relationship with MPA plasma levels (Figure 3B). The SHAP plot highlighted saliva concentration as the most influential predictor, followed by dose, urea, and PTP. These findings emphasize that incorporating saliva data significantly enhanced the model performance, offering predictive power beyond plasma measurements alone. Though relying on single-point saliva sampling may diminish the reliability of saliva-based pharmacokinetic assessment. Therefore, the potential of saliva as a supplementary monitoring tool should be approached with caution.

Additional limitations of the study included the small sample size of 76 patients, which may not reflect diverse PK responses. Furthermore, as the study involved stable renal transplant recipients in the late post-transplant phase, the findings may not be directly applicable to patients in the early post-transplant period or those with unstable graft function. Although separate PopPK models were developed for MMF and EC-MPS, integrating both formulations for comparative and ML analyses may introduce residual variability, as the two are not strictly bioequivalent despite normalization of MMF doses to EC-MPS equivalents using the standard 0.72 conversion factor. Furthermore, the pharmacokinetic analysis was based solely on trough (Cmin) concentrations, which limited the ability to estimate total MPA exposure (AUC) and explore more complex pharmacokinetic relationships. Future studies, including richer sampling designs, could enable AUC-based modeling and more comprehensive exposure assessment. Future evaluations with datasets, covering both pediatric and elderly patients, will further assess the applicability of the developed models.

Adding covariates like body weight, CrCl, albumin levels, and genetic polymorphisms, which were unavailable in this study due to its design as a clinical monitoring project, may enhance predictive ability. In particular, the absence of pharmacogenetic information, such as polymorphisms in UGTs, organic anion transport polypeptides, MRP2, IMPDH, and immune-response mediators, also represents a limitation, as these genes are known to influence MPA metabolism, efficacy, and toxicity, leading to variability in MPA levels and clinical outcomes [55]. Furthermore, renal function was assessed using available laboratory markers (creatinine and urea), as weight data were unavailable for GFR estimation. While this approach was appropriate for the available dataset, future studies could incorporate weight-independent formulas such as CKD-EPI 2021 to provide a more standardized evaluation of renal function.

Including pharmacodynamic data, such as immunosuppression markers, IMDPH inhibition rates, and cytokine levels, could improve dose personalization and predict outcomes by developing pharmacokinetic–pharmacodynamic (PKPD) models [20,56]. Larger datasets may support artificial neural networks and provide more precise predictions. Additionally, newly developed real-time ML models can be updated with new data to optimize dosing regimens in clinical decision support systems for immunosuppression, as has already been done in other medical fields [31,57]. Lastly, long-term assessment of PopPK-ML models is essential for evaluating effectiveness in dose personalization, graft acceptance vs. rejection rates, and preventing toxicity-related issues like infections and anemia.

While this study primarily concentrated on the pharmacokinetic and data-driven optimization of mycophenolate therapy, it is essential to recognize that clinical dosing decisions in transplantation go beyond PK considerations. Factors such as the individual risk of graft rejection, HLA matching, antibody status, donor type, and concomitant immunosuppressive therapy all influence dose selection and treatment outcomes. Furthermore, the clinical benefit of concentration-controlled vs. fixed-dose mycophenolate therapy remains uncertain, as demonstrated in trials such as OPTICEPT [58] and subsequent comparative studies [59]. Consequently, the present findings should be seen as pharmacokinetic insights that can complement, but not replace, broader clinical judgement in immunosuppressive management.

## 5. Conclusions

This study highlights the potential of combining PopPK modeling with ML techniques to improve the characterization of MPA pharmacokinetics and support individualized dosing considerations in renal transplant patients. Two PopPK models were developed for the two MPA formulations, and Monte Carlo simulations explored dosage adjustments under varying clearance conditions, suggesting possible optimized regimens for patients with reduced clearance. The applied ML methods enhanced understanding of factors influencing MPA exposure, with PCA and ensemble models confirming the importance of dose, urea, and post-transplantation time. Moreover, the strong correlation between saliva and plasma MPA concentrations indicates that saliva may represent a promising, non-invasive adjunct for therapeutic monitoring, although further validation is required. Overall, the findings provide a methodological basis for future research toward integrating ML-assisted PopPK modeling into individualized immunosuppressive therapy.

## Figures and Tables

**Table 1:** Demographics, clinical data, and pharmacotherapy information of the patients participating in this study.

Characteristic	n (%) or Median (IQR)
Demographics	
Number of patients (n)	76
MPA plasma samples (n)	209
MPA saliva samples (n)	65
Age (years)	51 (14)
Gender (Men, Women)	50 (65.8%), 26 (34.2%)
Clinical Characteristics	
Post-Transplantation Time (months)	70 (84.3)
Live Donor Transplant	54 (71%)
Deceased Donor Transplant	18 (24%)
Administered Formulation	
EC-MPS	63 (82.9%)
MMF	13 (17.1%)
Laboratory Values	
White Blood Cells (10^9^/L)	7.9 (2.6)
Red Blood Cells (10^12^/L)	4.7 (0.9)
Hemoglobin (g/L)	138 (32)
Hematocrit (%)	41.4 (8.9)
Platelets (10^9^/L)	225 (88)
Urea (mmol/L)	7.8 (5.4)
Creatinine (μmol/L)	136 (60)

**Table 2:** Parameter estimates for the final population pharmacokinetic model of Mycophenolic acid (MPA) following the administration of the Enteric-Coated Mycophenolate Sodium (EC-MPS) formulation.

Parameter	Value	Standard Error	Relative Standard Error (%)
Fixed Effects			
kapop	0.18	0.03	15.7
Vpop	192.42	36.18	18.8
Clpop	9.3	0.78	8.34
β(PTP)	0.16	0.04	24.7
β(TDD)	0.77	0.15	19.2
Standard Deviation of the Random Effects			
ω(ka)	0.36	0.08	21.2
ω(V)	0.52	0.20	38.8
ω(Cl)	0.27	0.06	20.9
γ(ka)	0.28	0.14	48.3
γ(V)	0.52	0.15	29.2
γ(Cl)	0.31	0.04	11.2
Residual Error Model			
a	0.04	0.01	26
b	0.06	0.02	24.6

Key: a, the constant component of the residual error model; b, the proportional component of the residual error model; β(PTP), a factor representing the relationship between Cl and the time post-transplantation; β(TDD), a factor representing the relationship between Cl and total daily dose; Cl, clearance; Clpop, population mean of Cl; γ(Cl), interoccasion variability for Cl; γ(ka), interoccasion variability for ka; γ(V), interoccasion variability for V; ka, absorption rate constant; kapop, population mean of ka; ω(Cl), between-subject variability for Cl; ω(ka), between-subject variability for ka; ω(V), between-subject variability for V; V, volume of distribution; Vpop, population mean of V.

**Table 3:** Population parameter estimates for the final population pharmacokinetic model of Mycophenolic acid (MPA) following the Mycophenolate Mofetil (MMF) formulation administration.

Parameter	Value	Standard Error	Relative Standard Error (%)
Fixed Effects			
kapop	0.23	0.052	22.6
Vpop	196.43	56.768	28.9
Clpop	9.3	1.042	11.2
β(PTP)	0.33	0.072	21.8
β(TDD)	1.27	0.293	23.1
Standard Deviation of the Random Effects			
ω(ka)	0.27	0.072	26.5
ω(V)	0.09	0.029	32.4
ω(Cl)	0.32	0.061	19.2
γ(ka)	0.48	0.191	39.8
γ(V)	0.33	0.071	21.4
γ(Cl)	0.27	0.060	22.3
Residual Error Model			
b	0.17	0.064	37.5

Key: b, the proportional component of the residual error model; β(PTP), a factor representing the relationship between Cl and the time post-transplantatio; β(TDD), a factor representing the relationship between Cl and total daily dose; Cl, clearance; Clpop, population mean of Cl; γ(Cl), interoccasion variability for Cl; γ(ka), interoccasion variability for ka; γ(V), interoccasion variability for V; ka, absorption rate constant; kapop, population mean of ka; ω(Cl), between-subject variability for Cl; ω(ka), between-subject variability for ka; ω(V), between-subject variability for V; V, volume of distribution; Vpop, population mean of V.

## Data Availability

The data presented in this study are available on reasonable request from the corresponding author. The data are not publicly available due to ethical and privacy restrictions, as they contain sensitive clinical information from renal transplant patients.

## Abbreviations

The following abbreviations are used in this manuscript:

−2LL	−2 log likelihood
AIC	Akaike Information Criterion
ANN	Artificial Neural Networks
AUC_0–12_	Area Under the Concentration curve from zero to 12 h
BIC	Bayesian Information Criterion
CL	Clearance
CNIs	Calcineurin Inhibitors
CrCl	Creatinine Clearance
EC-MPS	Enteric-Coated Mycophenolate Sodium
EHR	Enterohepatic Recirculation
GFR	Glomerular Filtration Rate
Hb	Hemoglobulin
Ht	Hematocrit
IIV	Inter-individual variability
IMPDH	Inosine Monophosphate Dehydrogenase
IOV	Inter-occasion variability
ka	Absorption rate constant
ML	Machine Learning
MMF	Mycophenolate Mofetil
MPA	Mycophenolic Acid
MPAG	Mycophenolic Acid Glucuronide
MSE	Mean squared error
NLME	Nonlinear mixed-effects
NPC	Numerical predictive check
OATP	Organic anion transport polypeptide
PCA	Principal Component Analysis
PK	Pharmacokinetic
PKPD	Pharmacokinetic–Pharmacodynamic
MRP2	Multidrug resistance-associated protein 2
PLT	Platelets
PopPK	Population Pharmacokinetic
PTP	Post-Transplant Time
RBC	Red Blood Cells
RF	Random Forest
RSE	Relative standard error
SHAP	SHapley Additive Explanations
SVM	Support Vector Machines
TDD	Total Daily Dose
UGT	Uridine diphosphate glucuronosyltransferase
Vd	Volume of distribution
VPC	Visual predictive check
WBC	White Blood Cells
